# Impact of the 2023/24 Influenza Vaccination on Patients with Inflammatory Rheumatic Disease in Germany: Insights from a Nationwide, Longitudinal, Self-Reported Study

**DOI:** 10.3390/vaccines14020136

**Published:** 2026-01-29

**Authors:** Karolina Gente, Benedikt Ditz, Eike Bormann, Nadine Al-Azem, Gerd R. Burmester, Salma Charaf, Christian Fräbel, Gabriele Gilliam-Feld, Natalie Klüser, Anna Knothe, Ulf Müller-Ladner, Johannes Roth, Hendrik Schulze-Koops, Christof Specker, Mirko Steinmüller, Konstantinos Triantafyllias, Rebecca Hasseli

**Affiliations:** 1 Internal Medicine V, Hematology, Oncology and Rheumatology, Heidelberg University Hospital, 69120 Heidelberg, Germany; 2 Elisabeth-Klinik Bigge, 59939 Olsberg, Germany; 3 Institute of Biometry and Clinical Research, University Münster, 48149 Münster, Germany; 4 Section of Rheumatology and Clinical Immunology, Department of Internal Medicine D, University Hospital Münster, Albert-Schweizer-Campus 1, 48149 Münster, Germany; 5 Department of Rheumatology and Clinical Immunology, Charité-Universitätsmedizin Berlin, 10117 Berlin, Germany; 6 Department of Internal Medicine I, University Hospital Giessen, 35392 Giessen, Germany; 7 German League Against Rheumatism, 53111 Bonn, Germany; 8 Department of Rheumatology and Clinical Immunology, Campus Kerckhoff, Justus-Liebig-University Giessen, 61231 Bad Nauheim, Germany; 9 Institute of Immunology, University of Münster, 48149 Münster, Germany; 10 Division of Rheumatology and Clinical Immunology, Medical Clinic and Policlinic IV, LMU University Hospital, 80539 Munich, Germany; 11 Department of Rheumatology and Clinical Immunology, Evangelisches Krankenhaus Kliniken Essen-Mitte, 45136 Essen, Germany; 12 Rheumatology Practice, 35578 Wetzlar, Germany; 13 Department of Rheumatology, Acute Rheumatology Center Rhineland-Palatinate, 55543 Bad Kreuznach, Germany; 14 Department of Internal Medicine I, Division of Rheumatology and Clinical Immunology, University Medical Center of the Johannes Gutenberg University, 55131 Mainz, Germany

**Keywords:** vaccination, immune-mediated inflammatory disease, immunomodulation, flu, immunosuppressants

## Abstract

**Background:** Patients with inflammatory rheumatic diseases (IRD) are susceptible to influenza infections and their complications. However, they may avoid vaccination for fear of exacerbating their IRD. This study evaluates the 2023/24 influenza vaccine in IRD patients, aiming to provide recommendations for this group in the upcoming season. **Methods:** In this prospective, longitudinal study, we assessed the self-reported impact of influenza vaccination on patients with IRD. Participants were recruited nationwide between October and December 2023 and completed an online questionnaire after vaccination as well as at three and six months of follow-up. **Results:** Among 633 patients, 87.5% were female, with a median age of 50.4 (18–84) years. Post-vaccination, 50% experienced injection site pain; 41% reported no side effects. IRD flares occurred in 5%, with 1% requiring changes to immunomodulation. Among 428 patients with follow-up, influenza infections were reported in 38 patients (8.9%), including 10 (2.3%) with reinfections. No severe cases requiring hospitalization were reported. Spondyloarthritis patients had higher susceptibility to influenza (*p* = 0.002), accounting for 55.3% of infections. IRD flare-ups in the 12 months before vaccination predicted infections (*p* = 0.002). **Conclusions:** The 2023/24 vaccine was well tolerated by IRD patients, with no impact on the course of the disease in 95% of cases. Only 9% of patients reported influenza infections, none of which were severe. In light of these findings, physicians are advised to recommend vaccination to eligible IRD patients prior to or in the respective season.

## 1. Introduction

Vaccines are among the most effective measures against infectious diseases, yet rising skepticism and misinformation about vaccines and their adjuvants contribute to declining global vaccination rates. No test currently guarantees individual vaccine effectiveness, despite extensive research efforts in this area. While immunogenicity assays, including serologic, neutralization, and cellular tests, indicate immune response, they do not guarantee clinical protection [1,2,3,4].

Influenza vaccination is an effective and affordable preventive strategy. In Germany, the Standing Committee on Vaccination (STIKO) recommends influenza vaccination for patients with inflammatory rheumatic diseases (IRD) on immunosuppressive therapy, with high-dose or adjuvanted vaccines being preferred for individuals aged ≥ 60 years [5]. IRD, like rheumatoid arthritis (RA) and systemic lupus erythematosus (SLE), are linked to an increased risk of influenza infections and complications [1]. Increased susceptibility is due to underlying rheumatic diseases and immunosuppressive therapies used for treatment [6,7]. Furthermore, influenza infections can trigger disease flares in patients with IRD. In SLE, influenza is significantly associated with increased incidence of flares during the first week post-infection, often leading to hospitalization [8].

Given the significant risks associated with influenza infections in patients with IRD, vaccination can effectively reduce the risk of respiratory morbidity and mortality in this population [9]. However, data on influenza vaccine efficacy and safety in IRD remain limited, raising concerns among both patients and physicians. Moreover, the routine exclusion of patients with IRD from vaccine approval trials further contributes to the uncertainty surrounding this issue.

Given these considerations, evaluating the effectiveness and tolerability of influenza vaccination in patients with IRD is essential. This study provides self-reported longitudinal data on these aspects of the 2023/2024 vaccine in IRD. We aim to offer recommendations for this group during the upcoming season to enhance vaccination uptake and address safety and efficacy concerns in this population.

## 2. Methods

### 2.1. Study Design and Participants

From 1 October to 31 December 2023, influenza patients receiving the 2023/24 influenza vaccine were invited to participate in a six-month longitudinal survey assessing vaccine effectiveness and tolerability. The inclusion criteria were: (i) age of at least 18 years, (ii) self-reported IRD diagnosis, (iii) influenza vaccination during the study period, and (iv) informed consent. Follow-up surveys were conducted three and six months after vaccination. The follow-up surveys inquired about outcomes occurring between 0–3 months (after the initial survey) and >3–6 months (since the previous survey).

An online questionnaire (www.RheumaVir.de, accessed on 20 December 2025) was developed with two sections.

(a) Demographics: this section collected data on location, age, gender, comorbidities, IRD type, immunomodulatory or immunosuppressive treatment, disease activity, and vaccination history.

(b) Influenza Vaccination: This section assessed participants’ vaccination experiences, its impact on disease activity, confidence in vaccines, perception of risk, and willingness to vaccinate in the future (see Supplementary Materials). The vaccine brand was not assessed. Based on previous studies indicating that patients are often unable to reliably report this information, we decided not to include it in the survey.

### 2.2. Data Collection

Survey promotion included posters with QR codes, social media, patient organization channels, and direct emails. Representatives from the German League Against Rheumatism were involved throughout the study. Informed consent was obtained before survey access. Participation was voluntary and uncompensated.

The study followed STROBE guidelines for reporting observational research.

### 2.3. Outcome Measures

The primary outcomes were the frequency and duration of disease flares following the 2023/2024 influenza vaccination. Secondary outcomes included the rate and progression of influenza infections in vaccinated IRD patients, stratified by IRD subtypes.

### 2.4. Statistical Analysis

Categorical variables are presented as absolute and relative frequencies, and continuous variables as median and range. Group comparisons were performed using χ^2^, Fisher’s exact, or Kruskal–Wallis tests, depending on variable type and cell sizes. Patients with no, one, or two influenza infections were compared using Fisher’s exact tests for categorical variables and Kruskal–Wallis tests for continuous variables. Wilcoxon–Mann–Whitney tests were used to compare disease activity between two groups. Statistical analyses were conducted using SPSS Statistics (IBM SPSS Statistics, V.26.0) and SAS 9.4 (SAS Institute, Cary, NC, USA).

### 2.5. Ethics Approval

The study was conducted in accordance with the principles of the Declaration of Helsinki and approved by the ethics committee of the Justus-Liebig-University Giessen (#AZ 19/23, 7 March 2023), Germany. Patient consent for publication was not required according to ethics committee. Informed consent was obtained from all subjects involved in the study.

## 3. Results

### 3.1. Patient Characteristics and Baseline Data

A total of 633 patients participated (median age 50.4 years, 87.5% female; Table 1), with rheumatoid arthritis as the most common IRD at 49.8%. Arterial hypertension was the most common comorbidity (26.5%); 41% reported no comorbidities. Two-thirds were treated in rheumatology practices, 28% in hospital outpatient departments, and 6% without rheumatologist care. More than half received glucocorticoids, and 43% were on biologics. Most patients (85.8%) continued their IRD therapy during vaccination; 9.2% discontinued on a physician’s advice, and 5.1% stopped independently (Table 2). Influenza vaccination at baseline was co-administered with another vaccine in 22.4% of cases, primarily coronavirus disease 2019 (COVID-19) vaccines (20.1%), followed by pneumococcal or shingles vaccination (n = 6, 1.0%).

### 3.2. Vaccination Ever History

Prior to the current influenza vaccination, participants reported the following previous vaccinations: Nearly all participants reported a previous COVID-19 vaccination (92.6%; Table 3). More than half received over four COVID-19 vaccines, 30% received at least three, and 2.8% two. Previous pneumococcal vaccination was reported in 64.9%, followed by measles (41.7%), shingles (40.1%), tuberculosis (15.3%), and respiratory syncytial virus (RSV, 4.3%). Only 2.8% reported no prior vaccinations.

### 3.3. Tolerability of Influenza Vaccination in IRD Patients

Half of the patients (50.1%) reported pain at the injection site after baseline vaccination, followed by fatigue (20.7%). 5.2% of patients reported an IRD flare post-vaccination. Consequently, four patients (0.6%) were either switched to a new IRD therapy or their existing therapy was supplemented with an additional IRD-related medication (Table 4). In follow-up surveys, 10.8% of the patients reported new onset of vaccine-related side effects since the last survey after three months, and 6.8% after six months. Over 90% of participants expressed willingness to receive an influenza vaccination during the next flu season.

### 3.4. Occurrences of Influenza Infection After 2023/2024 Influenza Vaccination and the Impact of the Vaccine on Disease Activity in IRD Patients

No severe influenza infections requiring hospitalization were reported. Among 428 patients with complete data, 38 (8.9%) experienced influenza infections, and 10 (2.3%) were infected twice (Table 1). Influenza infections were more common in patients with spondyloarthritides—including psoriatic, axial, and enteropathic spondyloarthritides—than in patients with other IRD types (55.3% vs. 26.1%; *p* = 0.002, Table 1). Notably, a greater proportion of men than women had spondyloarthritides (42.6% vs. 24.4%; *p* = 0.000334).

While age, treatment, and comorbidities did not significantly differ among patients with zero, one, or two infections, IRD flare-ups in the 12 months before vaccination were significant predictors of infections: the median duration of IRD flares was significantly longer in infected patients (45 days, range 0–365) than in non-infected patients (10 days, range 0–365; *p* = 0.002, Table 1). Subanalyses for IRD subtypes partially confirmed this observation: in patients with RA, those infected with influenza reported a higher number of days with flares in the past year compared to uninfected patients (72 ± 78.8 vs. 36.3 ± 66.3 days; *p* = 0.034). A similar trend was observed in patients with spondyloarthritides, who reported even more days with flares, aligning with the higher total infection rate compared to other IRD types (90.2 ± 103.9 vs. 53.8 ± 82.8 days; *p* = 0.018). In contrast, no significant differences were found in patients with connective tissue diseases or concurrent fibromyalgia (Table 5).

### 3.5. Source of Information, Reasons for Receiving Influenza Vaccination, and Concerns

Over half of the participants (n = 370, 58.5%) reported receiving information about influenza vaccination from a physician. Additionally, 23% of the participants (n = 145) referred to various sources such as newspapers, radio, television, online news, social media, and personal online research.

Participants reported several personal reasons for receiving the influenza vaccination, including: (i) belonging to a risk group (n = 533, 84.2%), (ii) the desire to avoid influenza infection (n = 471, 74.4%), (iii) the intention to protect others (n = 175, 27.7%), (iv) employment in the healthcare sector (n = 99, 15.6%), (v) work in critical environments such as schools (n = 58, 9.2%), and (vi) other reasons (n = 28, 4.4%).

Most patients (n = 495; 78.2%) expressed no concerns about the influenza vaccination. The remaining participants reported varying levels of concern: mild (n = 101, 16.0%), moderate (n = 32, 5.1%), and only a small proportion reported high (n = 4, 0.6%) and very high concern (n = 1, 0.1%). Identified concerns included (i) fear of side effects (n = 91, 14.4%), (ii) worries about increased disease activity post-vaccination (n = 64, 10.1%), (iii) perceptions of insufficient efficacy of the vaccination (n = 28, 4.4%), (iv) concerns about long-term side effects (n = 20, 3.2%), (v) potential genomic consequences (n = 6, 1.0%), and (vi) insufficient information related to the vaccination (n = 5, 0.8%). These findings underscore the importance of addressing patient concerns while highlighting the overall effectiveness and tolerability of the influenza vaccination in individuals with IRD.

## 4. Discussion

In this nationwide study involving over 600 IRD patients in Germany, the 2023/2024 influenza vaccine was well tolerated, with only 8.9% of the patients experiencing influenza infection. No hospitalizations, major disease flares, or severe adverse events were reported. These observations were preserved despite ongoing treatment with disease modifying anti-rheumatic drugs (DMARDs) in the majority of patients, including approximately one-fifth who received concurrent vaccinations.

Retrospective data from 30,788 IRD patients indicate that influenza vaccination in DMARD recipients reduces the incidence of influenza-like illnesses, COPD exacerbations, and pneumonia-related hospitalizations and mortality [9]. Our prospective cohort study confirms these observations, especially concerning the preventative effect of vaccinations on influenza-associated hospitalizations.

It is encouraging that over 90% of participants expressed willingness to receive vaccination in the next season. However, despite clear benefits, many patients remain hesitant and opt out of vaccination. Previous studies identified several key barriers to vaccination uptake among IRD patients, including lack of physician recommendations, younger age, concerns about vaccine safety and efficacy, fear of disease flares, limited awareness or misconceptions regarding vaccination benefits, and mistrust of the medical system or pharmaceutical industry [10,11,12].

Concerns that vaccination may exacerbate underlying IRD and doubts about efficacy due to immunosuppressive therapy were particularly confirmed in our survey. Nakafero et al. found no significant association between inactivated influenza vaccination and rheumatoid arthritis flares, glucocorticoid prescriptions, fever, or new-onset vasculitis. Vaccination was even associated with reduced primary care consultations for joint pain within 90 days post-vaccination [13]. Consistent with these findings, our prospective study observed no significant IRD flares following vaccination (Table 4).

The concern about flares, ranked second in the survey, can be reassuringly addressed by physicians with minimal time investment. In this context, the absence of a direct recommendation from a rheumatologist or general practitioner consistently appears as the most significant predictor of non-vaccination [10,11,12]. However, only 58.5% of participants reported receiving vaccination information from a physician. In contrast, 94% of patients received regular rheumatologist care, highlighting a significant communication gap where rheumatologists fail to address vaccination, which urgently needs improvement. Differing from other studies, limited access to vaccination services and lack of awareness of cost-free vaccines likely play a minor role in the insufficient vaccination rates among IRD patients in Germany [10,11,12,14].

While immune responses may vary by disease type and DMARD treatment, only one participant receiving rituximab—a biological, immunosuppressive agent used primarily for severe RA and systemic IRDs known to cause B-cell depletion—reported influenza in our study [15]. Unexpectedly, participants with connective tissue diseases and other IRD types associated with higher infection susceptibility reported influenza infections less frequently than those with spondyloarthritis. This observation may be linked to previously reported higher infection rates in patients with elevated disease activity prior to vaccination, as also observed in our spondyloarthritis patients [16]. In our study, patients with spondyloarthritis had more days with flares prior to vaccination, aligning with the higher total infection rate compared to other IRD types (Table 5). In contrast, no significant differences were found in patients with fibromyalgia (n = 10) and connective tissue diseases (n = 11) (Table 5). Notably, the statistical test may not have been significant for fibromyalgia because of a smaller sample size. However, it is crucial to consider that sex differences may have influenced the observed outcomes in spondyloarthritides, as men are generally more susceptible to infectious diseases and show weaker vaccine-induced humoral responses than women [17].

To our knowledge, this is the first study to prospectively evaluate the effectiveness of influenza vaccination in reducing infection severity in IRD patients in real-world setting, including reinfection cases. Reassuringly, the reinfection rate of 2.3% falls within the range previously reported in a general population, which varies from 1% in Queensland, Australia and 4% in Guangxi, China [18,19]. Therefore, vaccination has proven effective in preventing severe influenza cases, even in patients experiencing reinfection.

The study’s key strengths include: (i) a substantial sample size; (ii) homogenous data retrieved from a single healthcare system over a defined period, ensuring that all patients received the same standard of medical care and experienced the same influenza season; and (iii) data gathered through a standardized online survey.

This study has several limitations. Only vaccinated IRD patients were invited to participate, and recruitment supported by patient organizations may have introduced selection bias, favoring well-informed individuals who are willing to be vaccinated, as suggested by their higher uptake of other vaccines and reported willingness for future influenza vaccination. Given that this was a very select group of patients who opted to receive the influenza vaccine and enrolled in the study, the results should be considered with certain caution. The educational level and socioeconomic status of the participants were not documented, potentially influencing their knowledge and attitudes toward vaccination. The self-administered questionnaire may have excluded patients who experienced severe side effects or influenza infections from participation. Additionally, information on disease duration, prior medications, vaccine type, and other infectious diseases that could influence IRD flares or the risk of influenza was not recorded. A limitation of the study is that it was not possible to compare the efficacy and tolerability of the vaccine to those of a general population. This is due to the fact that only vaccinated patients and those who voluntarily chose to participate were included in the study.

## 5. Conclusions

The 2023/24 vaccine was well tolerated by IRD patients, with no impact on the course of the disease in 95% of cases. Only 9% of patients reported influenza infections, none of which were severe. Given the substantial benefits of influenza vaccination for IRD patients, physicians are advised to recommend it to eligible IRD individuals in the upcoming seasons and proactively address potential concerns regarding the influenza vaccination.

## Figures and Tables

**Table 1:** Data of all patients (n = 633). Non-infected patients (n = 380) were compared to once infected (n = 38) and twice infected (n = 10) patients. IRD = inflammatory rheumatic disease, ANCA = Anti-neutrophil cytoplasmic antibodies, IgG4 = immunoglobulin G4, csDMARD = conventional synthetic (cs) disease modifying antirheumatic drug (DMARD), bDMARD = biological disease-modifying antirheumatic drugs, ts = targeted synthetic, TNF-a = tumor necrosis factor alpha, IL = interleukin, GC = glucocorticoid.

	All Patients (n = 633)		No Influenza (n = 380)		Influenza Infection (Once, n = 38)		Influenza Infection (Twice, n = 10)		*p*-Value	Effect Size
**Patient characteristics**										
Age (years. median (range))	50.4 (18–84)		55 (18–82)		54 (18–73)		59 (27–73)		0.201	<0.0001
	n	%	n	%	n	%	n	%		
Gender (female)	554	87.5	338	89	34	97	8	80		
**Inflammatory rheumatic disease (IRD, multiple selection possible)**										
Rheumatoid arthritis	315	49.8	191	50.3	18	47.4	3	30	0.492	0.063
Spondyloarthritis (psoriatic arthritis, axial and enteropathic spondyloarthritis	170	26.9	99	26.1	21	55.3	0	0	0.002	0.151
Connective tissue diseases	178	28.1	102	26.8	6	15.8	5	50	0.079	0.086
ANCA-associated vasculitis	9	1.4	6	3.2	0	0	1	10	0.169	0.108
Other type of vasculitis (giant cell arteritis, polyarteritis nodosa, Behcet’s disease, IgG4-associated disease, all other type of vasculitis)	18	2.8	12	3.2	0	0	0	0	0.02	0.226
Sarcoidosis	12	1.9	7	1.8	0	0	0	0	1	0.46
Autoinflammatory diseases	6	1.0	3	0.8	1	2.6	0	0	0.38	0.056
Crystal-associated arthropathy	3	0.5	2	0.5	0	0	0	0	1	0.024
Other rheumatic diseases	10	1.6	7	1.8	0	0	0	0	1	0.046
Unknown which type of IRD	7	1.1	4	1.1	0	0	0	0	1	0.035
Concomitant fibromyalgia	70	11.1	45	11.8	9	23.7	1	10	0.119	0.101
Concomitant small fiber neuropathy	6	1.0	4	1.1	0	0	0	0	1	0.035
On how many days of the past 12 months did you have an increased disease activity (days. median. range)	15	0–365	10	0–365	45	0–365	8.5	2–150	0.002	0.0138
**IRD treatment (multiple selection possible)**										
Total glucocorticoids	189	29.9	103	27.1	11	28.9	3	30	0.92	0.015
Total csDMARD (methotrexate, leflunomide, sulfasalazine, cyclosporine A, hydroxychloroquine, azathioprine)	342	54.0	200	52.6	24	63.2	7	70	0.293	0.078
• Methotrexate	211	33.3	123	32.4	17	44.7	4	40	0.272	0.077
Total bDMARD (inhibitors of TNF-a, IL-1, -6, -12/23, -17 and others)	258	42.9	159	41.8	14	36.8	2	20	0.336	0.072
• TNF-a inhibition (etanercept, infliximab, adalimumab, certolizumab, golimumab)	154	24.3	96	25.3	9	23.7	1	10	0.665	0.054
• IL-17 inhibition (secukinumab, bimekizumab, ixekizumab)	16	2.5	8	2.1	0	0	0	0	0.709	0.06
• IL-12/23 inhibition (ustekinumab, risankizumab, guselkumab)	13	2.1	9	2.4	0	0	0	0	1	0.052
• IL-6 inhibition (tocilizumab, sarilumab)	28	4.4	18	4.7	0	0	1	10	0.238	0.078
• IL-1 inhibition (anakinra, canakinumab)	7	1.1	3	0.8	1	2.6	0	0	0.38	0.056
Total tsDMARD (upadacitinib, baricitinib, filgotinib, tofacitinib, apremilast)	57	9.0	31	8.2	4	10.5	1	10	0.559	0.026
Immunosuppressives (mycophenolate, rituximab)	37	5.9	26	6.8	1	2.6	0	0	0.738	0.045
Other immunomodulation (colchicine, immunoglobulins)	5	0.8	4	1.1	0	0	0	0	1	0.035
No immunomodulatory treatment	79	12.5	51	13.3	5	13.2	0	0	0.645	0.06
csDMARD in combination with bDMARD	84	13.3	68	17.9	9	23.7	0	0	0.241	0.084
csDMARD in combination with bDMARD and GC	24	3.8	20	5.3	2	5.3	0	0	1	0.036
csDMARD in combination with tsDMARD	15	2.4	7	1.8	2	5.3	0	0	0.349	0.071
csDMARD in combination with tsDMARD and GC	7	1.1	3	0.8	0	0	0	0	1	0.03
**Comorbidities**										
Cardiovascular disease	31	4.9	15	3.9	1	2.6	1	10	0.465	0.051
Arrhythmia	18	2.8	14	3.7	0	0	0	0	0.733	0.065
Arterial hypertension	168	26.5	103	27.1	10	26.3	3	30	1	0.011
Bronchial asthma	95	15.0	59	15.5	7	18.4	2	20	0.724	0.028
Chronic obstructive lung disease	7	1.1	3	0.8	3	7.9	0	0	0.025	0.173
Pulmonary hypertension	11	1.7	10	2.6	0	0	0	0	0.692	0.055
Lung fibrosis	32	5.1	19	5	0	0	1	10	0.209	0.078
Cancer (history/active)	14	2.2	7	1.8	1	2.6	0	0	0.617	0.027
Chronic renal failure			19	5	3	7.9	0	0	0.671	0.052
Liver fibrosis			0	0	0	0	0	0		
Osteoporosis	66	10.4	41	10.8	4	10.5	2	20	0.495	0.045
Diabetes mellitus	31	4.9	14	3.7	3	7.9	0	0	0.366	0.069
Depression	135	21.3	75	19.7	12	31.6	4	40	0.063	0.109
No relevant comorbidity	259	40.9	153	40.3	14	36.8	3	30	0.84	0.037

**Table 2:** Discontinuation of the inflammatory rheumatic disease (IRD) treatment during the administration of the influenza vaccination.

When Did You Discontinue Your IRD Treatment?	n	%
Up to 1 week before vaccination	37	5.8
1–2 weeks before vaccination	16	2.5
More than 2 weeks before vaccination	3	0.5
Up to 1 week after vaccination	36	5.7
1–2 weeks after vaccination	27	4.3
More than 2 weeks after vaccination	2	0.3

**Table 3:** Self-reported history of vaccines ever prior to influenza vaccination 2023/24.

History of Vaccination Prior to Influenza Vaccination 2023/24		
	n	%
COVID-19 vaccination	586	92.6
Pneumococcal vaccination	411	64.9
Measles vaccination	264	41.7
Shingles vaccination	254	40.1
Other vaccination	169	26.7
TBC vaccination	97	15.3
RSV vaccination	27	4.3
No prior vaccination	18	2.8
Unknown vaccination status	4	0.6

**Table 4:** Reported side effects of influenza vaccination and impact on the disease activity of the IRD reported in the first and follow-up surveys. Baseline survey was conducted immediately after influenza vaccination. In the follow-up survey after 3 months and between >3–6 months any changes since the last survey were asked. Regarding side effects, multiple choices were available. AE = adverse event, DSA = disease activity, IRD = inflammatory rheumatic disease.

	Start of the Study (n = 633)		3 Months After Vaccination (n = 536)		>3–6 Months After Vaccination (n = 428)	
**Side effects after administration of influenza vaccination**	n	%	n	%	n	%
No AE	261	41.2	478	89.1	399	93.2
New side effects since the last survey (follow-up surveys)			58	10.8	29	6.8
Pain at injection site	317	50.1	40	7.5	14	3.3
Fatigue	131	20.7	16	3.0	10	2.3
Myalgia, Arthralgia or backpain (other than pain due to disease activity)	99	15.6	8	1.5	11	2.6
Headache	86	13.6	8	1.5	4	0.9
Shivering	42	6.6	6	1.1	2	0.5
Fever	23	3.6	6	1.1	0	0
Other AE	19	3.0	6	1.1	0	0
Nausea	12	1.9	1	0.2	1	0.2
Diarrhea	7	1.1	3	0.6	2	0.5
Allergic reaction	6	1.0	0	0	2	0.5
Stomach pain	5	0.8	0	0	0	0
Vomiting	1	0.2	0	0	0	0
Duration of adverse event (days, median, range)	2 (0–16)		3 (0–6)		2 (1–15)	
** Disease activity ** (DSA)						
Increase of DSA immediately after vaccination	33	5.2				
• Time between vaccination and increase of DSA (days, median, range) • Level of DSA (0–10, median, range)	2 (0–40) 3 (1–8)					
• Increase of DSA since the last survey			97	18.1	82	19.6
• Level of DSA (0–10, median, range)			7 (1–9)		7 (2–9)	
• Duration of DSA (days, median, range)			6 (1–60)		5 (1–60)	
** Change of IRD treatment due to DSA **						
No	13	2.1	34	6.3	28	6.5
Yes, Prednisolone < 5 mg/day	12	1.9	32	6.0	25	5.8
Yes, Prednisolone > 5 mg/day	4	0.6	23	4.3	23	5.4
Yes, new IRD therapy	2	0.3	12	2.2	10	2.3
Yes, new concomitant IRD-related drug	2	0.3	3	0.6	8	1.9
**Would you get vaccinated against influenza again?** (only asked in the follow-up surveys)						
Yes	/	/	505	94.2	399	93.2
No	/	/	11	2.1	8	1.9
Unknown	/	/	20	3.7	12	2.8

**Table 5:** Data on the frequency with which disease flares were reported within the 12 months prior to influenza vaccination, categorized by type of inflammatory rheumatic disease and state of influenza infection. The mean values, standard deviations, medians, and ranges are shown. Mann–Whitney test was used to evaluate the results. The calculated *p*-value is shown.

Days of Disease Flares Within the Past 12 Months Prior to Influenza Vaccination			*p*-Value
	**Rheumatoid Arthritis**		
Mean (days, range)	No Influenza Infection (n = 191) 36.3 ± 66.3 (0; 365)	Influenza Infection (n = 21) 72 ± 78.8 (0; 250)	*p* = 0.034
Median (days, range)	12 (0; 365)	30 (0; 250)	
	**Spondyloarthritis (including psoriatic arthritis, axial and enteropathic spondyloarthritis)**		*p* = 0.018
	No influenza infection (n = 99)	Influenza infection (n = 21)	
Mean (days, range)	53.8 ± 82.8 (0; 365)	90.2 ± 103.9 (2; 365)	
Median (days, range)	21 (0; 365)	50 (2; 365)	
	**Connective tissue diseases**		*p* = 0.593
	No influenza infection (n = 102)	Influenza infection (n = 11)	
Mean (days, range)	34.3 ± 67.2 (0; 365)	30.2 ± 45.6 (0; 150)	
Median (days, range)	10 (0; 365)	10 (0; 150)	
	**Concomitant fibromyalgia**		*p* = 0.164
	No influenza infection (n = 45)	Influenza infection (n = 10)	
	66.0 ± 96.0 (0; 365)	91.9 ± 106.9 (0; 365)	
	30 (0; 365)	62.5 (0; 365)	

## Data Availability

All data relevant to the study are included in the article.

## Supplementary Materials

The following supporting information can be downloaded at: Online Questionnaire: https://www.mdpi.com/article/10.3390/vaccines14020136/s1. The online questionnaire used for this study is composed of two parts: a baseline questionnaire and a follow-up questionnaire. The questions have been translated into English.
