# Supplementary material for: Impact of the 2023/24 Influenza Vaccination on Patients with Inflammatory Rheumatic Disease in Germany: Insights from a Nationwide, Longitudinal, Self-Reported Study
Source: Vaccines (Basel). 2026 Jan 29;14(2):136. doi: 10.3390/vaccines14020136 (PMC12945301; doi:10.3390/vaccines14020136)
Supplement: Supplementary file 1 [file vaccines-14-00136-s001.zip › vaccines-4101376-supplementary.pdf]

Information about the patient	
Age	Year of birth: ____________
Gender	[]female []male [] divers
In which federal state are you located?	[] Baden-Württemberg<br>[] Bavaria<br>[] Berlin<br>[] Brandenburg<br>[] Bremen<br>[] Hamburg<br>[] Hesse<br>[] Mecklenburg-Western Pomerania<br>[] Lower Saxony<br>[] North Rhine-Westphalia<br>[] Rhineland-Palatinate<br>[] Saarland<br>[] Saxony<br>[] Saxony-Anhalt<br>[] Schleswig-Holstein<br>[] Thuringia
Which disease do you suffer from? (multiple choice possible)	[] Rheumatoid arthritis<br><br>**Spondylarthritis**<br>[] Axial spondyloarthritis (Bechterew's disease)<br>[] Psoriatic arthritis (psoriasis arthritis)<br>[] Enteropathic arthritis (arthritis in ulcerative colitis/Crohn's disease)<br><br>**Connective tissue diseases**<br>[] Polymyositis/dermatomyositis/inclusion body myositis (muscle inflammation)<br>[] Sjögren's syndrome<br>[] Systemic lupus erythematosus<br>[] Systemic sclerosis (scleroderma)<br>[] Mixed collagenosis<br>[] Overlap collagenosis<br><br>**Vascular inflammation with detection of ANCA antibodies**<br>[] Granulomatosis with polyangiitis (GPA, Wegener's disease)<br>[] Eosinophilic GPA (Churg-Strauss vasculitis)<br>[] Microscopic polyangiitis<br><br>**Other types of vascular inflammation**<br>[] Polymyalgia rheumatica (polymyalgia)<br>[] Large vessel inflammation (giant cell arteritis, Takayasu, temporal arteritis)<br>[] Panarteritis nodosa (Kussmaul-Maier disease)

	[] Behcet's disease<br>[] IgG4-associated vasculitis<br>[] Other vascular inflammation: ___________________<br><br>[] Sarcoidosis<br>[] Fever syndromes (e.g. Still's disease)<br>[] Gout<br>[] Pseudogout (chondrocalcinosis)<br><br>[] Fibromyalgia syndrome<br>[] Small fibre neuropathy<br><br>**[] other disease not mentioned,**<br>Name of the disease: _________________<br>[] I do not know which disease I have
Which of the following medications do you take for your rheumatic disease? (multiple choice possible)	[] Cortisone<br>[] Methotrexate (e.g. Lantarel, Metex, Trexjet)<br>[] Azathioprine (e.g. Immurek)<br>[] Ciclosporin A (e.g. Sandimmun)<br>[] Voclosporin (Lupkynis)<br>[] Leflunomide (e.g. Arava)<br>[] Hydroxychloroquine (e.g. Quensyl)<br>[] Sulfasalazine (e.g. Pleon RA)<br>[] Colchicine (Colchicum)<br><br>[]Baricitinib (Olumiant)<br>[] Tofacitinib (Xeljanz)<br>[] Upadacitinib (Rinvoq)<br>[] Filgotinib (Jyseleca)<br><br>[] Adalimumab (e.g. Humira, Amgevita, Imraldi, Hyrimoz)<br>[] Infliximab (e.g. Remicade, Remsima, Flixabi)<br>[] Certolizumab (e.g. Cimzia)<br>[] Golimumab (e.g. Simponi)<br>[] Etanercept (e.g. Benepali, Erelzi, Enbrel)<br><br>[Secukinumab (Cosentyx)<br>[] Ixekizumab (Taltz)<br>[] Bimekizumab (Bimzelx)<br>[] Ustekinumab (Stelara)<br>[] Guselkumab (Tremfya)<br>[] Risankizumab (Skyrizi)<br><br>[] Tocilizumab (RoActemra)<br>[] Sarilumab (Kevzara)<br><br>[] Abatacept (Orencia)

	[] Rituximab (e.g. Mabthera, Truxima)<br>[] Anakinra (Kineret)<br>[] Canakinumab (Ilaris)<br>[] Belimumab (Benlyste)<br>[] Mycophenolate mofetil (CellCept)<br>[] Cyclophosphamide (Endoxan)<br>[] Immunoglobulins (e.g. Privigen, Kiovig, Intratec)<br>[] Apremilast (Otezla)<br>[] Mepolizumab (Nucala)<br>[] Anifrolumab (Saphnelo)<br><br>[] other medications not mentioned here: _________________<br><br>[] no medication (exclusion of other options)
How many days have you had a rheumatic flare-up in the last 12 months?	Number: 0-365 days
Do you have a rheumatologist?	[] No, I do not have a rheumatologist<br>[] Yes, I am being treated in a rheumatological practice/medical centre<br>[] Yes, I am being treated in a rheumatological outpatient clinic of a hospital
Do you suffer from any of the concomitant diseases listed below?<br>(multiple choice possible)	[] Cardiovascular disease (e.g. heart failure, CHD, bypass, heart attack)<br>[] Atrial fibrillation<br>[] High blood pressure<br>[] Bronchial asthma<br>[] Chronic obstructive pulmonary disease (COPD)<br>[] Pulmonary hypertension<br>[] Pulmonary fibrosis<br>[] Cancer (acute/history of cancer)<br>[] Chronic kidney disease<br>[] Liver failure<br>[] Osteoporosis<br>[] Diabetes mellitus<br>[] Depression
Have you been vaccinated against other pathogens (except flu vaccination)?<br>(multiple choice possible)	[] Pneumococcal vaccination (pneumonia vaccination)<br>[] Tuberculosis vaccination<br>[] RSV vaccination<br>[] Corona vaccination<br>- [] Number of vaccinations: 1[] 2[] 3[] 4[] 5[] more than 5[]<br>[] Measles<br>[] Shingles

	[] Other vaccination (free text)<br>[] no (exclude other information)<br>[] unknown (exclude other information)
Did you experience side effects with other vaccinations in the past? (multiple choice possible)	[] no [] yes: which vaccination? (see above)<br>Selection of vaccinations mentioned<br><br>If yes, which side effects?<br>[] Pain at the injection site<br>[] Fever above 38.5°C<br>[] Severe tiredness<br>[] Nausea<br>[] Vomiting<br>[] Chills<br>[] Headache<br>[] Abdominal pain<br>[] diarrhoea<br>[] Muscle, limb or back pain (different from a rheumatic attack)<br>[] Rheumatic attack<br>[] Allergic reaction<br>[] Other (free text)
**About the flu vaccination**	
When did you receive the flu vaccination?	[] Date: XX/XX/20XX (will be asked the first time, after that no longer necessary)
If you have received another vaccination at the same time as the flu jab	[] no [] yes<br><br>If yes, which vaccination?<br>[] Pneumococcal vaccination (pneumonia vaccination)<br>[] RSV vaccination<br>[] Corona vaccination<br>[] Shingles<br>[] Other vaccination (free text)<br>[] Yes, but vaccine unknown
How did you find out about the flu vaccination? (multiple choice possible)	[] personal environment<br>[] Newspaper<br>[] Radio<br>[] News on TV<br>[] News online<br>[] Social networks (e.g. Facebook, Twitter etc.)<br>[] Online research<br>[] via my doctor<br>[] via information events<br>[] I have not informed myself (exclude other information)
Why did you get vaccinated? (multiple choice possible)	[] Because I don't want to get seriously ill with the flu<br>[] Because I belong to the risk group<br>[] Because I work in the healthcare sector myself<br>[] Because I work in a critical area myself

	[] Protection of relatives/friends/patients<br>[] Other reasons (free text)
Do you have any concerns about the flu vaccination?	[] no concerns<br>[] few concerns<br>[] moderate concerns<br>[] many concerns<br>[] very many concerns<br><br>If there are (moderate/many/very/many) concerns:<br>Why do you have concerns?<br>[] Long-term effects of the vaccine<br>[] Changes to my own DNA (genetic material) as a result of the vaccination<br>[] no effect of the vaccination on me<br>[] Possible side effects<br>[] Rheumatism flare-up after the disease<br>[] I was not sufficiently informed
Have you paused or extended your medication before/after the vaccination?	[] No (please also select 'No' if you received the vaccination in between the administration of your medication so that there was no delay in your therapy)<br>[] Yes, paused on the recommendation of my rheumatologist<br>[] Yes, paused independently<br><br>If 'Yes, independently': (multiple choice possible)<br>For what reason?<br>[] Advice from friends/acquaintances<br>[] I have already done this with other vaccinations<br>[] out of fear<br>[] other reasons (free text)<br><br>If medication was paused (recommendation from rheumatologist & independently)<br>Did you pause your medication before the vaccination?<br>[] yes, days to 1 week before the vaccination<br>[] yes, 1-2 weeks before the vaccination<br>[] yes, longer than 2 weeks before the vaccination<br>[] no<br><br>Did you stop taking your medication after the vaccination?<br>[] yes, days to 1 week before the vaccination<br>[] yes, 1-2 weeks before the vaccination<br>[] yes, longer than 2 weeks after the vaccination<br>[] no
Did you experience any side effects after the flu vaccination (other than rheumatic flare up)?	[] no[] yes<br><br>If yes, which side effects occurred (multiple answers)?<br>[] Pain at the injection site<br>[] Fever above 38.5°C

	[] Severe tiredness<br>[] Nausea<br>[] Vomiting<br>[] Chills<br>[] Headache<br>[] Abdominal pain<br>[] diarrhoea<br>[] Muscle, limb or back pain (different from a rheumatic flare up)<br>[] Allergic reaction<br>[] Other (free text)<br><br>If yes:<br>How many days did/have the side effect(s) last?<br>______ Days
Did you experience a rheumatic flare up after the vaccination?	[] yes[] no<br><br>If yes:<br>How many days after vaccination did a flare up occur?<br>[] _____________ days after the vaccination<br><br>If yes:<br>How severe was the rheumatic flare-up?<br>(Scale 1-10; 1: very mild, 10: very severe)<br><br>If yes:<br>How many days did the rheumatic flare up last?<br>[] _____________ Days<br><br>If yes:<br>Did you have to change your rheumatism medication due to the rheumatic flare up?<br>[] no<br>[] yes, only slight changes (e.g. increase in prednisolone by 5 mg/day, temporary use of anti-inflammatory pain medication such as ibuprofen, diclofenac, naproxen, etoricoxib, celecoxib)<br>[] Yes, I had to increase the prednisolone (cortisone) by more than 5 mg/day<br>[] Yes, I have been given a new medication<br>[] Yes, I have been given another medication in addition to the existing one
**Follow-up (3 and 6 months after influenza vaccination)**	
Did any new side effects occur after the last survey following the flu vaccination that were not mentioned in	[] no[] yes<br><br>If yes, which side effects occurred (multiple answers)?<br>[] Pain at the injection site<br>[] Fever above 38.5°C

the first survey (apart from rheumatism)?	[] Severe tiredness<br>[] Nausea<br>[] Vomiting<br>[] Chills<br>[] Headache<br>[] Abdominal pain<br>[] diarrhoea<br>[] Muscle, limb or back pain (different from a rheumatic attack)<br>[] Allergic reaction<br>[] Other (free text)<br><br>If yes:<br>How many days after vaccination did you experience side effects?<br>[] _____________ days after the flu vaccination
Have you been infected with flu viruses despite having the flu vaccination?	[] no[] yes<br><br>If yes, when (date):<br><br>If yes:<br>What symptoms did the flu cause?<br>[] Fever<br>[] Chills<br>[] Cough<br>[] Sputum when coughing<br>[] Sore throat<br>[] cold<br>[] Muscle pain<br>[] Exhaustion<br>[] Headache<br>[] Shortness of breath<br>[] Dizziness<br>[] Abdominal pain<br>[] Diarrhoea<br>[] Vomiting<br>[] Nausea<br>[] less/hardly any appetite<br>[] loss of smell<br>[] loss of taste<br>[] cardiac arrhythmia<br>[] other symptoms ______________________________<br>[] no symptoms occurred (exclude other information)<br><br>Did you have to be hospitalised because of the flu?<br>[] no<br>[] yes, without oxygen administration<br>[] yes, with oxygen administration

Has there been another rheumatic flare up since the last enquiry after the flu vaccination? (if another rheumatic flare up has occurred in the meantime)	[] yes[] no<br><br>If yes:<br>How many days after vaccination did you have an episode?<br>[] ____________ days after flu vaccination<br><br>If yes:<br>How severe was the rheumatic attack?<br>(Scale 1-10; 1: very mild, 10: very severe)<br><br>If yes:<br>How many days did the rheumatic attack last?<br>[] ____________ Days<br><br>If yes:<br>Did you have to change your medication due to the rheumatic flare-up?<br>[] no<br>[] yes, only slight changes (e.g. increase in prednisolone by 5 mg/day, temporary use of anti-inflammatory pain medication such as ibuprofen, diclofenac, naproxen, etoricoxib, celecoxib)<br>[] Yes, I had to increase the prednisolone (cortisone) by more than 5 mg/day<br>[] Yes, I have been given a new medication<br>[] Yes, I have been given another medication in addition to the existing one
Have you received another vaccination against other pathogens (apart from the flu vaccination) since the last survey?	[] no (exclude other information)<br>[] Pneumococcal vaccination (pneumonia vaccination)<br>[] Tuberculosis vaccination<br>[] RSV vaccination<br>[] Corona vaccination<br>[] Measles<br>[] Shingles<br>[] Other vaccination (free text)<br>[] unknown (exclude other information)
Would you have the flu vaccination again?	[] yes<br>[] no<br>[] I do not know
